# Global Research Trends in Cervical Cancer Survival in the Last Two Decades: A Bibliometric Analysis

**DOI:** 10.21315/mjms2024.31.6.8

**Published:** 2024-12-31

**Authors:** Syed S Abrar, Suhaily Mohd Hairon, Najib Majdi Yaacob, Mohd Pazudin Ismail, Seoparjoo Azmel Mohd Isa

**Affiliations:** 1 Department of Community Medicine, School of Medical Sciences, Universiti Sains Malaysia, Kelantan, Malaysia; 2 Unit of Biostatistics and Research Methodology, School of Medical Sciences, Universiti Sains Malaysia, Kelantan, Malaysia; 3 Department of Obstetrics and Gynaecology, School of Medical Sciences, Universiti Sains Malaysia, Kelantan, Malaysia; 4 Department of Pathology, School of Medical Sciences, Universiti Sains Malaysia, Kelantan, Malaysia

**Keywords:** cervical cancer, survival, bibliometric, VOSviewer

## Abstract

Cervical cancer poses a significant challenge to global health, particularly in low- and middle-income countries. Tracking the survival rates of cancer patients through data gathered by population-based cancer registries constitutes a crucial aspect of cancer management. In recent years, attention within bibliometric studies has been directed towards examining the correlation between inflammation and radiotherapy in cervical cancer. However, to date, there is no published literature investigating research trends in cervical cancer survival through bibliometric analysis. Thus, this study was undertaken to identify and analyse global research patterns and hotpots in this area. A systematic search was performed within the Web of Science Core Collection (WoSCC) database, employing the following search parameters: TITLE - (((survival) OR (survival rate) OR (survival analysis) OR (survival probability)) AND ((cervical cancer) OR (cervical carcinoma))). A comprehensive analysis of research trends was conducted utilising various tools on the WoSCC website and VOSviewer. A total of 840 papers pertaining to cervical cancer and survival were identified from 45 distinct departments or subjects. Notably, the People’s Republic of China and the USA collectively accounted for half of all publications from 2000 to 2023. An extensive cohort comprising 4,759 authors affiliated with 1,454 institutions across 82 countries contributed to the progression of this research domain. Despite a substantial increase in research on cervical cancer survival over the last decade, it is essential to encourage and conduct research, particularly in high-risk regions, especially in countries classified as low- or middle-income.

## Introduction

Cervical cancer poses a significant challenge to global health, particularly with an increasing occurrence in low- and middle-income countries. It is the most prevalent cancer affecting female reproductive organs worldwide (1). The majority of cancer-related deaths among women in Africa and Central America are attributed to cervical cancer (1). According to the American Cancer Society, the widespread adoption of screening substantially contributed to a more than 50% reduction in cervical cancer incidence rates between the mid-1970s and mid-2000s. However, in the past decade, these rates have stabilised. Notably, from 2012 to 2019, there was a 1.7% yearly increase in rates among women aged 30–44 years (2). The age-standardised incidence of cervical cancer in 2020 surpassed the threshold set by the WHO’s Cervical Cancer Elimination Initiative in 173 countries (3).

Approximately 84% of global cervical cancer cases and 88% of cervical cancer-related deaths are reported in developing countries (4). In developed nations, such as the USA and England, the 5-year survival rates are 67% and 60%, respectively (5, 6). Conversely, in developing countries like Thailand, the 5-year survival rate is under 60% (7).

As per the European Cancer Information System, a decline in both the incidence and fatality rates of cervical cancer has been observed in several European Union nations. Notably, patients diagnosed with cervical cancer between 2000 and 2007 exhibited the highest 5-year survival rates in Western Europe, while the lowest rates were recorded in various Eastern European countries (8).

A comprehensive meta-analysis involving 45 studies and 16,122 cervical cancer patients in Africa revealed a 5-year survival rate of 41% and a disease-free survival (DFS) rate of 66%. Notably, Northern Africa demonstrated a 25% higher 5-year overall survival (OS) compared to other sub-Saharan countries. The central zone exhibited the lowest OS at 24%, while the eastern, western and southern zones reported 5-year OS rates of 32%, 35% and 49%, respectively (9).

In Asian cervical cancer patients, a systematic review and meta-analysis reported a 1-year survival rate of 76.62%, with 3-, 5- and 10-year rates of 68.77%, 62.34% and 61.60%, respectively. Taiwan had the lowest and Kuwait had the highest 10-year survival rate (10). For individuals under 50 years in India, the 5-year survival rate was 76.0%, and survival rates for different stages were 83.5% for Stage I, 80.6% for Stage II, 66.0% for Stage III and 37.1% for Stage IV (11). In Iran, a study reported a 35.5% 5-year survival rate for women with cervical cancer (12).

Bibliometric analysis has gained popularity in the recent decade, as it helps in evaluating academic papers and identifying the latest research trends on a given topic. Recently, some bibliometric studies have focused on cervical cancer and radiotherapy (13) and inflammation in cervical cancer (14). Another bibliometric analysis explored cervical cancer trends in sub-Saharan Africa (15). However, to date, no published literature has investigated global research trends in cervical cancer survival. Therefore, the current study was conducted to explore and analyse global trends in cervical cancer survival using the Web of Science Core Collection (WoSCC) database.

## Methods

### Search Strategy

**Figure 1:**
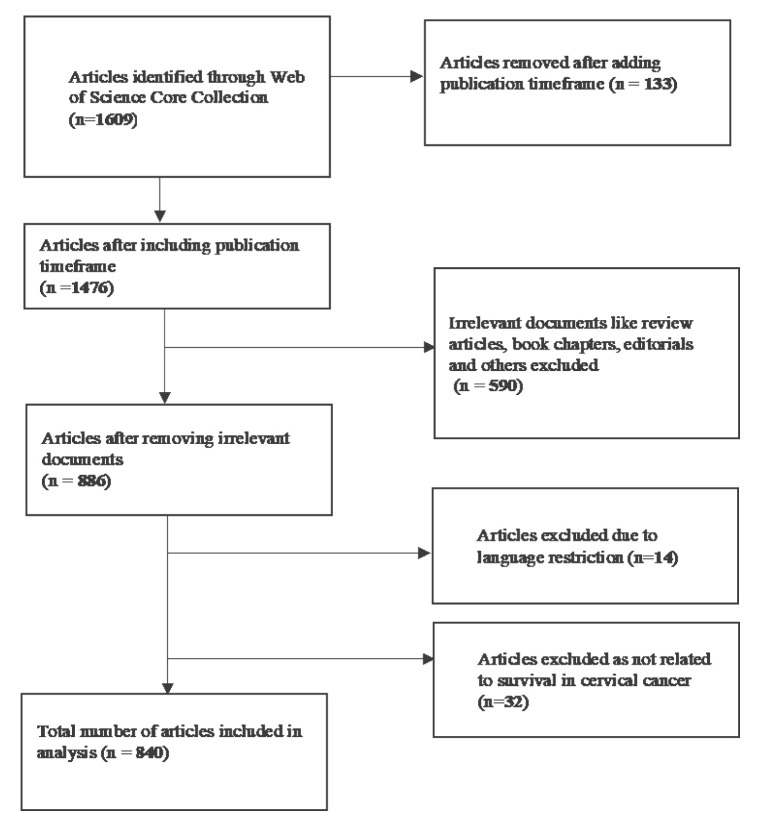
Flowchart of data collection of survival in cervical cancer publications in WoSCC database

For this study, a search on the WoSCC database was conducted on 25 April 2024 using the following search terms: TITLE - (((survival) OR (survival rate) OR (survival analysis) OR (survival probability)) AND ((cervical cancer) OR (cervical carcinoma))) (Figure 1). WoSCC, a premier database encompassing scholarly journals, books and proceedings published since 1970, incorporates ESCI (Emerging Sources Citation Index), SSCI (Social Science Citation Index) and SCIE (Science Citation Index Expanded).

The search yielded a total of 1,609 publications using the specified search terms. After refining the publication timeframe (01-01-2000 to 12-31-2023), 1,476 publications met the eligibility criteria for further analysis. Most authors predominantly utilised English as their language of choice, accounting for 872 published articles (98.41%). Additionally, there were 8 articles (0.90%) in Spanish, 2 articles (0.225%) each in French and Portuguese, and 1 article (0.11%) each in German and Russian. By restricting the language to ‘English’ and excluding irrelevant documents, such as review articles, book chapters, editorials, meeting abstracts, proceeding papers and others, 840 publications were included in the final analysis.

### Data Collection

The following details were obtained from each article: article titles, author names, number of authors, publication year, institution affiliations, countries, journal names, thematic categories, article sources, keywords and citation counts.

### Data Analysis

Various tools on the WoSCC website were utilised to analyse trends in the number of publications over the years, pinpoint the most referenced documents and determine institutions with the highest publication rates. To further analyse and generate network visualisations, VOSviewer (version 1.16.20) was employed, utilising different methods, such as co-occurrences of keywords and bibliographic coupling of sources.

## Results

### Publication Output

**Figure 2:**
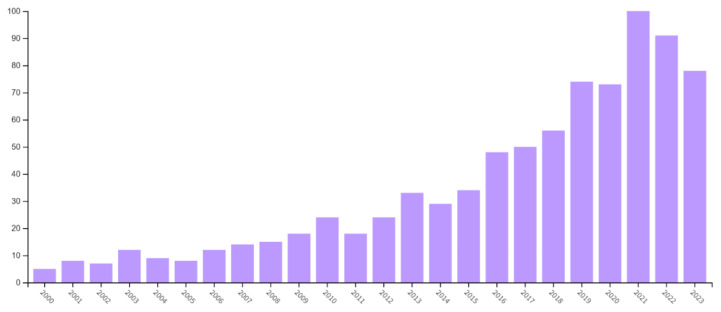
Growth of publication output between 2000 and 2023

Analysing the number of publications each year revealed notable growth in cervical cancer survival research over the past decade. The number of publications increased from 132 in 2000–2010 to 708 in 2011–2023, indicating a significant surge in the latter period. The highest number of publications was recorded in 2021 (11.90%). The analysis encompassed 840 publications, revealing a growing trend. A total of 4,759 authors affiliated with 1,454 institutions across 82 countries contributed to the advancement of this research field. Figure 2 shows the growth in publication output over the last 23 years.

### Authors, Institutions and Countries

**Figure 3:**
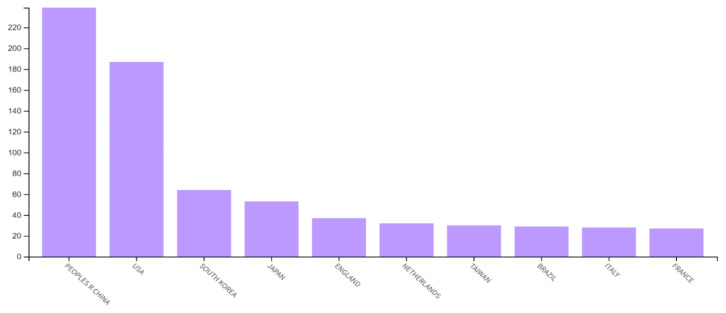
The top 10 countries with the highest number of publications

Regarding countries with the most publications, the People’s Republic of China led with 28.452%, followed by the USA (22.262%), South Korea (7.691%), Japan (6.301%) and England (4.405%). China and the USA collectively contributed to half of all literature. The countries with the fewest publications were mostly low- or lower-middle-income countries. Figure 3 depicts the top 10 countries and their publication output.

**Figure 4:**
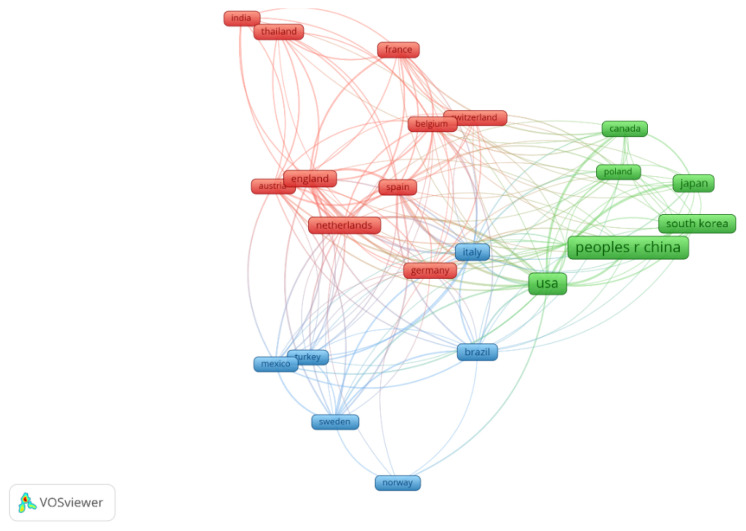
Collaborative network between countries

Next, we used VOSviewer to filter and depict 24 countries with 10 or more publications and constructed a collaborative network based on the quantity and interrelation of publications within each country (Figure 4). According to the VOSviewer map, countries situated closer to each other exhibited a higher degree of relatedness. Conversely, the thickness of the connecting lines indicates the strength of the links among the countries. Remarkably, there is extensive collaboration between various countries. For instance, the USA engages in close cooperation with 22 countries, with maximum collaboration occurring with Japan and Canada. Similarly, Japan and China collaborate actively with other regions. The USA had the highest link strength of 90, followed by Spain with 72, England with a link strength of 65 and the Netherlands with 56. The maximum number of collaborations took place between the USA and Japan, followed by the USA and Canada and the USA and China. Although China produced a high number of publications, the number of collaborative partnerships with other nations was low.

The top 15 institutions are distributed across three countries, with two-fifths of them situated in China. Among these, the institutions that produced the most publications were the Chinese Academy of Medical Sciences, Peking Union Medical College, the University of Texas System and Sun Yat Sen University. Koji Matsuo and Jinghe Lang emerged as the most active authors in this field, with 11 documents (314 citations) and 10 documents (105 citations) each. Following closely behind are Seiji Mabuchi, Chunlin Chen and Ping Liu, with 9 publications each. The most widely cited authors were Bradley J. Monk, Krishnasu S. Tewari and Mario M. Leitao, with 1,321, 1,278 and 1,244 citations each.

### Publishers, Journals and Citation Analysis

**Figure 5:**
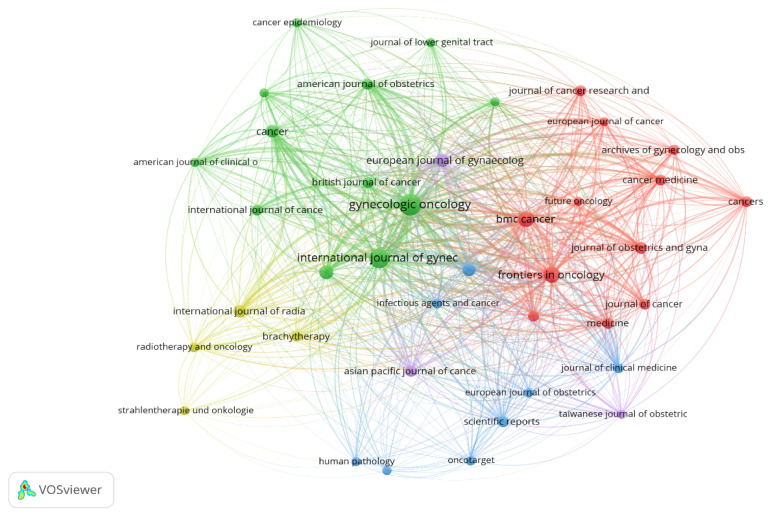
Bibliographic coupling of sources

In terms of publishers, Elsevier ranked highest, with 191 publications, followed by Springer Nature, Wiley and Lippincott Williams & Wilkins with 120, 69 and 54 publications, respectively. All other publishers had fewer than 50 publications. The top five journals with the most publications were: *Gynecologic Oncology*, *International Journal of Gynecological Cancer*, *Frontiers in Oncology*, *BMC Cancer* and *PLOS ONE*. The journals with the most citations were: *Gynecologic Oncology* (2,566), *International Journal of Gynecological Cancer* (1,095), *Cancer* (1,054), *Radiotherapy and Oncology* (609) and *International Journal of Gynecological Cancer* (587). The bibliographic coupling of sources was obtained when 39 journals were included in the analysis, with each having at least five documents (Figure 5). Each circle represents a journal, with its size indicating the number of publications in that journal according to the bibliographic coupling analysis. The larger the circle, the higher the number of publications. The lines between the circles indicate connections between journals, while the differently coloured connection networks signify clusters of cooperation among different journals. Each colour represents a distinct cluster. The analysis resulted in five clusters comprising a total of 39 items.

The top-cited papers are presented in Table 1. Of the 5 papers with the most citations, one was published in *Lancet*, which has an impact factor of 168.9, and another was published in the *New England Journal of Medicine*, which has an impact factor of 158.5 (16, 17). The remaining three papers were published in *Cancer Research*, *Cancer* and *Radiotherapy and Oncology* (18–20). The impact factor of these journals ranged from 11.2 to 5.7. A total of 83 publications had no citations.

### Thematic Categories and Keywords

Additionally, 840 papers addressing cervical cancer and survival were published across 45 distinct departments or subjects. The highest number of articles originated from the following research areas: oncology, obstetrics gynaecology, radiology nuclear medicine medical imaging, general internal medicine and public environmental occupational health.

**Figure 6:**
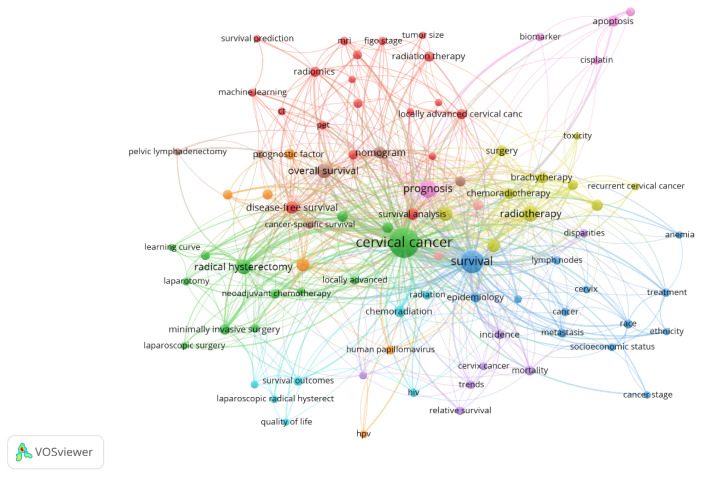
A network map illustrating trending author keywords

Figure 6 shows the co-occurrences of author keywords. Of the 1,393 keywords, 88 met the threshold with a minimum of 5 occurrences each. The most common keywords were ‘cervical cancer’, ‘survival’ and ‘prognosis’, with 489, 186 and 82 occurrences each. The five keywords with the highest link strength were ‘cervical cancer’, ‘survival’, ‘prognosis’, ‘radical hysterectomy’ and ‘radiotherapy’. The least-used keywords were ‘uterine cervical neoplasm’, ‘tumour size’, ‘survival prediction’, ‘adjuvant radiotherapy’ and ‘cancer stage’, among others.

## Conclusion

To the best of our knowledge, this is the first bibliometric examination exploring global research trends in cervical cancer survival.

The current bibliometric analysis of publication data spanning 2000–2023 reveals a discernible trend in the literature on cervical cancer survival research. Initially, there was a gradual increase in publications, followed by a notable acceleration in the rate of publications in subsequent years.

In the current analysis, 82 nations were identified, with 78 of them having fewer than 50 publications on cervical cancer and survival research. Notably, research from low- or lower-middle-income countries is limited. This emphasises the critical need for an escalation in research efforts focused on cancer survival within these specific regions. Tracking the survival rates of cancer patients through data gathered by population-based cancer registries constitutes a crucial aspect of cancer management. According to the World Health Organization (WHO), cervical cancer ranks as the second most common cancer in low- and middle-income countries, constituting approximately 85% of all new cases worldwide. Mortality rates in low- and middle-income countries are 18 times higher than those in high-income countries (1). Consequently, it is imperative to delve further into the underlying reasons for the limited output of intervention research in these regions and subsequently enhance research productivity.

Additionally, nearly 50% of publications did not include any details regarding the funding agencies and almost 58% made no mention of grants. The scarcity of grants poses a significant obstacle to conducting high-quality research. Researchers should be informed about the diverse funding agencies that exist to support cervical cancer research and to enhance their awareness of available resources and opportunities.

### Advantages and Limitations

The current analysis offers several advantages. First, it represents the inaugural systematic bibliometric analysis of survival in cervical cancer, providing comprehensive recommendations for researchers engaged in related studies. Second, the utilisation of an employed bibliometric tool, like VOSviewer, enhances the objectivity of our data analysis methodology. Compared to traditional reviews, bibliometric analysis provides a more thorough understanding of research hotspots and frontiers. Finally, the various analyses performed in the current study have generated essential information like research focal points, appropriate journals, trending keywords and ongoing research patterns, which can be beneficial to upcoming and existing researchers. They can review the current trends in research related to cervical cancer survival and understand the research landscape. Furthermore, they can also conduct their own analyses and contribute to addressing existing gaps in the field.

However, it is crucial to acknowledge several limitations. First, the present study focused on publications in English, which may have introduced selection bias. Second, reliance on articles exclusively from the WoSCC database may lead to the omission of relevant references in the bibliography. Third, citation metrics are influenced by time, making recent articles prone to fewer citations than earlier publications due to their publication date.

Additionally, it is important to note that the present research adopted a quantitative approach. A more thorough and comprehensive qualitative evaluation of the papers focused on specific subjects should be considered for deeper understanding and insight. Combining quantitative and qualitative analyses could provide a more well-rounded perspective on the researched topics. Awareness of these limitations is essential for a nuanced interpretation of the findings.

## Recommendations

With the continued rise in cervical cancer cases, it is crucial to promote and conduct research, particularly in high-risk regions, notably in countries classified as low- or middle-income. Integrating a systematic review is suggested to gain deeper insights into trends in global cervical cancer survival research.

## Figures and Tables

**Table 1:** The top most cited publications in cervical cancer survival research

Author	Title	Country	Total no. of citations	Citations in 2023	Average citation per year	Journal	Impact factor
Tewari et al. (16)	Improved survival with bevacizumab in advanced cervical cancer	USA	859	62	78.09	*New England Journal of Medicine*	158.5
Walenta et al. (17)	High lactate levels predict likelihood of metastases, tumor recurrence, and restricted patient survival in uman cervical cancers	Germany	662	57	26.48	*Cancer Research*	11.2
Sturzda et al. (18)	Image guided brachytherapy in locally advanced cervical cancer: improved pelvic control and survival in RetroEMBRACE, a multicenter cohort study	Austria	483	60	53.67	*Radiotherapy and Oncology*	5.7
Tewari et al. (19)	Bevacizumab for advanced cervical cancer: final overall survival and adverse event analysis of a randomised, controlled, open-label, phase 3 trial (Gynecologic Oncology Group 240)	USA	353	60	44.13	*Lancet*	168.9
Kidd et al. (20)	The standardized uptake value for F-18 fluorodeoxyglucose is a sensitive predictive biomarker for cervical cancer treatment response and survival	USA	244	5	13.56	*Cancer*	6.2
